# Bodily obsessions: intrusiveness of organs in somatic obsessive–compulsive disorder

**DOI:** 10.1007/s11019-022-10090-3

**Published:** 2022-05-26

**Authors:** Joni P. Puranen

**Affiliations:** University of Jyväskylä, Jyvaskyla, Finland

**Keywords:** Attention, Phenomenology, Obsessive–compulsive disorder, Somatic obsessions, Breathing

## Abstract

In this paper, I will provide a phenomenological analysis of somatic obsessions at times present in obsessive–compulsive disorder. I will compare two different types of bodily obsessions, which have a different neurological-physiological underpinning: anguishing awareness of one’s own heartbeat and of one’s own breathing. In addition, I will contrast these two with how one experiences one’s own liver. I will use the concepts "tactility obsessions” and "motility obsessions”, which I have coined for the purpose of this comparison. In other words, these are obsessions concerning the felt sense of one’s autonomous organs and obsessions concerning one’s ability to voluntarily move. Ultimately, I claim that the core lived experience in somatic obsessive–compulsive disorder should not only be understood as having to do with intruding and "distorted thoughts” concerning bodily processes, but could also be understood as having to do with a felt sense of our organs interrupting and intruding our daily lives.

## Introduction

What happens to (or in) “me”, if “I” cannot trust the vegetative automaticity of my body? What happens, if I “get stuck” on breathing or blinking “consciously”? If such is the case and if I do feel trapped in paying attention to my breathing, blinking or to my heartbeat, then to what exactly am I attending to? Am I attending to the movement of some specific muscles? Am I attending to my attention or attentiveness itself as a process? Or to an experience of suffocation, if I do not consciously continue maintaining the circulation of air? Or, am I attending to my thoughts concerning these particular bodily processes? How does it feel when a bodily process overtakes my whole life?

We can all recognise the phenomena of becoming annoyingly aware of our beating hearts or the slow and rhythmic movement of breathing, when, e.g., we are having a spell of transitional insomnia (or maybe when we read a paper such as this one on somatic obsessions in obsessive–compulsive disorder), but such an awareness of one’s own body usually does not last for long. For a few of us, however, such episodes can become increasingly frequent, time consuming, uncomfortable or even downright unbearable. In this paper, I provide an analysis of the experiential and “subjectively” lived bodily dimensions of a psycho-pathological condition called “somatic^1^ obsessive–compulsive disorder”.^2^ I provisionally describe this condition as an anguishing and inhibiting hyper-awareness^3^ of one’s own body in its functions, but in addition to the emotive or practical dimensions of the condition my analysis also illuminates its temporal, spatial, sensory, and motor aspects. Somatic OCD, thus, can be understood as an abnormality of body awareness.

I will compare two different types of bodily obsessions, which have a different anatomical underpinning: anguishing awareness of one’s own heartbeat and of one’s own breathing. I will contrast these two types of abnormal experiences with our common condition of *not* experiencing our own inner organs, e.g., liver, kidneys or reproductive glands. I will use the concepts “tactility obsessions” and “motility obessions” to refer to obsessive experiences one’s autonomous organs and obsessions concerning one’s ability to volitionally move one’s body, which I have coined for this purpose. Ultimately, I claim that the core lived experience in somatic obsessive–compulsive disorder does not exclusively entail “distorted thoughts” (Wegner 1989) concerning bodily processes, as is proposed by Hershfield and Corboy in one of the few textual descriptions of somatic OCD (2013, p. 181), but can also, or better, be understood as concerning felt sense of our organs interrupting and intruding both our “here and now” and our future.

My following presentation of the current understanding of somatic OCD cannot be exhaustive concerning the whole literary corpus on obsessive–compulsive disorder,^4^ but, as far as I am aware, up to this point there exists no research on the lived bodily experiences of somatic OCD and hardly any research on somatic OCD in general. Therefore, my analysis of the lived experience of somatic OCD and my suggestion of distinguishing between tactility and motility obsessions, will contribute to the effort to develop our understanding of this hidden, disinhibiting and intriguing disorder and could also prove to be useful for diagnostic and therapeutic practices.

## Background and methodology

Today, much attention is given to the possible health effects of practices that aim to *increase* one’s awareness their own body, such as mindfulness (Didonna 2008; Alper 2016), cognitive behavioural therapy (Wells and Fisher 2015) or mindfulness-practices in medicine (Buchholz 2015; Chung 2015; Ludwig and Kabat-Zinn 2008). Attention itself is also studied in great detail in the clinical sciences (Tang and Posner 2013; Velden and Roepstorff 2015; Tang et al. 2015). Most of this literature and research points at possible health benefits from increased attention or awareness of one’s own body and its processes. So far, however, little attention has been paid to phenomena or instances, such as described in the opening lines of this paper, in which increased attention of one’s body might be detrimental to one’s health, and can even result in, or accompany, severe anxiety and prolonged suffering.

In my analysis, I look at the surprisingly sparse psychological (Keuler 2011) and therapeutic (Hershfield and Corboy 2013) literature on the symptoms, reports and descriptions of how somatic OCD is experienced and I critically engage the role of body in these descriptions. To do so, I draw from the analyses of the visceral body undertaken by philosophers Jean-Luc Nancy and Drew Leder in their pioneering works on the topic. More specifically, I utilise Nancy’s concept of *intruder*, which is helpful for understanding the experience of intrusiveness felt and reported in somatic obsessions and Leder’s conceptualisation of *visceral inability,* which sheds light on the nature of volitional motility (and immotility) of the viscera.

My paper offers an analysis of the structural elements constitutive to lived bodily experiences of somatic OCD. In the phenomenologically oriented research into how experiences of one’s own body are structured, much attention has recently been paid to phenomenas such as dysappearance (Groven et al. 2013; Slatman 2016) and disappearance (Zeiler 2010) of one’s body in a variety of bodily changes. My analysis of somatic OCD ties to these discussions and more generally to experiential analyses of the body and corporeality. I add, however, a new line of analysis by discussing somatic OCD as an abnormal and inhibiting variation of body self-awareness. This is a novel contribution in the field of experiential philosophy of embodiment and brings completely new phenomena into theoretical discussions.

My study is broadly phenomenological in the sense that I analyse the appearance, disappearance and transparency of visceral organs and visceral processes in terms of how they are constituted *in* and *as* bodily experiences. More specifically, my methodological and conceptual starting points are in the philosophical exchange between Jean-Luc Nancy and Martin Heidegger, concerning existence and corporeality. I draw mainly from Nancy’s philosophical analysis of how bodies open a *site*, a *here* or *this place* for existing. Bodies offer a *place* for sensing, moving, talking, thinking and, generally speaking, for all the singular ways of being in the world. Nancy describes his position on bodies in *Corpus* (15) as follows:

Bodies are places of existence, and nothing exists without a place, a *there*, a ‘here,’ a ‘here is,’ for a *this*.

The novelty of Nancy’s *Corpus* is in how he allows us to think of existence and being in terms of sensing, sensitive and sensual bodies. In this paper, I examine somatic OCD as one particularly anguishing way of experiencing one’s body, when parts or areas in one’s “own” body become an intrusive and strange.

## Obsessive–compulsive disorder in DSM-5

Obsessive–compulsive disorder (OCD) is a chronic and quite often debilitating disorder.^5^ OCD is currently (according to F42 in DSM-5) diagnosed following four criteria. Firstly, there needs to be a presence of either “obsessions” or “compulsions” or of both.^6^ “Obsessions” are defined as recurring and persistent thoughts, urges or images that are experienced as disturbing, intrusive or unwanted, and which the individual attempts to ignore, suppress or neutralise through performing acts that are called “compulsions”. “Compulsions” are defined as repetitive behaviours (e.g. hand washing, ordering, checking) or mental acts (e.g. counting, wishing, praying, repeating certain words, sentences or mantras), which the individual feels obliged to perform in response to their obsessions, in order to prevent or reduce their anxiety or mental anguish. Yet these behaviours are not causally connected “in a realistic way” with what they are supposed to neutralise or prevent, or are clearly excessive. Secondly, obsessions or compulsions are taken to be time consuming or to cause a clinically significant suffering, anguish or impairment, be that social, occupational or related to other important areas of life. Thirdly and fourthly, medical and physiological (substance or drug abuse, etc.) causes and other mental disorders need to be ruled out.

In the somatic dimension of OCD (or in the somatic “spectrum”, “type” or “subtype”),^7^ one’s “awareness of” or their “attentiveness towards” their own bodily processes becomes something that bothers them significantly and causes them great anguish. Keuler (2011) describes the lived experience of somatic OCD as follows:

In a typical scenario, individuals begin to selectively attend to their swallowing, for example, and become anxious that they will become unable to *stop* thinking about their swallowing. Attempts to distract themselves fail, leading to higher levels of anxiety. This anxiety perpetuates the focus on swallowing, leaving them preoccupied and frustrated by their unsuccessful attempts to shift attention elsewhere.

The following bodily processes have been reported as being the most common *foci* for people suffering from somatic OCD: breathing, blinking, salivation and swallowing, body positioning, tactile sensations such as the heartbeat or itching, tinnitus, “eye floaters” and other visual distractions (e.g. seeing the profile of one’s own nose in their peripheral vision).^8^ Common compulsions in somatic OCD, as suggested by Keuler (2011) and Hershfield and Corboy (2013), consist mostly of failed attempts to turn one’s attention away from one’s obsessions, seeking help from medical professionals, seeking information regarding their condition, and ruminating on the fact that their abnormal awareness of their own bodies is ruining their lives. The question of how to clearly distinguish between obsessions and compulsions in the experiential dimensions of somatic OCD is beyond the scope of this paper.

## Experience of impairment in somatic OCD

The criterion of “significant impairment” reveals two details peculiar to the experience of somatic OCD. As Keuler (2011) notes, most people have experienced transient episodes of bodily hyper-awareness at some point, which would be classified as “non-significant” in terms of the diagnostic scale of suffering. This, I argue, has two important consequences for our analysis.

Firstly, if a diagnosis comes down to how impairing or intrusive one’s awareness of their own body is and if most of us have experienced transient episodes of excessive body-awareness, then the lived experience of the disorder should be available to philosophical reflection into structural features inherent to lived experience of one’s organs and their functions. In what follows, I examine breathing and heartbeat in terms of their tactile sensibility and volitional motility. Secondly, if such is the case—if most people recognise themselves as having experienced transient episodes identical in structure to those that are reported in instances of somatic OCD—then the condition might be far more common than what we expect and what our current understanding would lead us to believe.

This paper’s central claim is that the experience of somatic OCD is necessarily a tactile and on some occasions also a motile experience, in addition to being an experience concerning distorted thoughts, rumination or fears concerning particular bodily processes occurring in one’s own body. In the analysis that follows, I will focus on how the body is experienced in three bodily processes that have to do with the viscera. Two of these processes have been reported as being common *foci* in somatic OCD, whereas the third process does not appear in descriptions of the disorder and will be employed to provide contrast. These processes are: (i) breathing with lungs, (ii) a beating heart and (iii) a metabolising liver. I have chosen these three exemplary processes because their differing experiential tactility and visceral motility allow me to examine the experience of somatic OCD in terms of differences regarding their innervation and, furthermore, to study it in as an experience structured by corporeal automaticity, volitional motility and tactile sensibility of the visceral organs.

In order to distinguish the different manners in which our intestinal organs may “appear” to us, I will start by looking into to Jean-Luc Nancy’s analysis of visceral *intrusiveness* (and concealment) in “The intruder” (found in *Corpus* 2008, pp. 161–170) followed by Drew Leder’s analysis of visceral *motility* in *The Absent Body* (1990).

## Tactility and motility of the visceral body

In this section, I explicate two ways that we experience our visceral organs. Firstly, visceral organs are experienced tactilely as “intrusive” surfaces, movements or areas and secondly, they are experienced in regards to whether or not their processes or movements are “available” for our volitional movement. In order to further develop these conceptualisations I will turn to philosophers Jean-Luc Nancy and Drew Leder and their analyses of the visceral body.

The main goal for Nancy in his “The Intruder” is to show how a “self”, one’s “own” body, various parts and areas of that body, various processes and organs in bodies, transplants and grafts, contracting muscles and titanium screws are all intrusive and intruding; they are intrusive to one another, to themselves and to thought that ponders bodies. Nancy’s analysis of “his own” deteriorating heart (and his subsequent heart transplant) follows right on the footsteps of his deconstructive, ontological or “post-phenomenological” work on the “ontology of the body” undertaken in his pivotal work *Corpus*. For Nancy’s bodies in *Corpus*, classical phenomenological concepts such as *body intentionality*, *care*, *consciousness*, *ego, operative intentionality*, *self, subject*, *subjectivity* are either inwardly projected representations or imprinted significations emanating outward from unexamined or bypassed bodies. For Nancy, aforementioned philosophical, psychological or theological interpretations, formulated in terms of interiority and exteriority, fail in granting bodies their weight, their extendedness, exposure, sensibility and their sensuality, because they examine bodies as subsequent to spirit or mind (2008, pp. 67–73). Rather, for Nancy (2008, p. 15), bodies are *places* of being; bodies open singular places of being in the world—of being *here*.

How should we understand experiences of an autonomous and automatic organs calling for our attention from the dark visceral depths of our bodies? In his essay “The Intruder” Nancy describes how he senses his own deteriorating heart—how it becomes “an intruder” in “his own” body. At first, Nancy’s heart is intrusive, because it goes unnoticed.^9^ It is concealed like the soles of one’s feet while walking or a liver secreting bile in one’s abdomen. Nancy writes: “[I]t was strange by virtue of not being even perceptible, not even being present.” (2008, p. 163). To be more specific: mostly his heart is strange because it does not “call” for his attention by being tactilely perceivable through palpitations, movement, pain or discomfort. Initially, Nancy’s heart is “silent”, if we remember René Leriche’s^10^ famous definition of health as the silence of the organs.

But how should we understand organs in terms of their tactile availability? In his essay “On The Soul”, Nancy (2008, p. 129) writes:

[H]ealth is life in the silence of the organs, when I don't sense my stomach, my heart, or my viscera. There's an intimacy there, but an intimacy that is merely not there, not sensible, it's of the order of the mass.

As we can read, Nancy describes the transparency or concealment of an unnoticed organ with the concept of “mass” (*masse*). And what is “mass” in this context of sensing bodies? Nancy defines “mass” in the following way: a sensing body feels its own extendedness and its exposure. This means that sensing bodies feel themselves as touching and as touched, whereas a mass does not extend, touch nor stand available for touch. In *Corpus* Nancy (124) writes: “W]hat isn't body is mass, or substance in the sense of mass, without extension, without exposition, a point.” Nancy’s definition of mass as non-extending, and thus as something that is not available “to” or “for” touch, is precisely how our internal organs are usually given to us: we do not notice them, because they do not appear as tactile, moving, painful areas, surfaces or organs. Their “felt” sense, their sensibility, is concentrated to (or beyond) an absolute minimum of a “point”. This means that, in effect, they disappear from our awareness and become part of the unavailable background of our bodily being.^11^ Therefore, we have two descriptions of the visceral concealment and correspondingly of visceral intrusion. For Leriche, an unnoticed heart is metaphorically silent; Leriche describes how our organs (can potentially) call for us.^12^ For Nancy, an unnoticed heart is without extension and exposition and therefore does not touch us nor appear available for touch. Nancy’s account highlights the role of touch in how we notice our organs, whereas Leriche’s description, if understood metaphorically, gives organs the ability to cry or shout in pain.

Nancy continues by describing (2008, p. 162) his slightly increasing distress: at times he feels palpitations, minor irregularities and breaks in the rhythm. Nevertheless, these concerns mainly live on the screen of a monitor or in the language spoken by doctors and between doctors. Later on, Nancy’s failing heart begins to “defect” from him. In other words, the silence of his heart is about to change and his heart is becoming something he cannot ignore. Nancy describes (2008, p. 163) this change as follows:

It became strange to me, intruding by defection: almost by rejection, if not by dejection. I had this heart at the tip of my tongue, like improper food. Rather like heartburn [*un haut-le-coeur*], but gently.

When his heart turns against him, it becomes articulated by becoming an intrusive organ demarcated by pain. With this gentle pain he feels his heart touching him, which *is* himself touching himself. His heart becomes intrusive in a way similar to how one might describe acid regurgitation or a foreign object in one’s mouth. Nancy continues by describing (2008, p. 163) a change in how he senses himself as a stranger in his own body, when his heart intrudes him:

[S]omething broke away from me, or this thing surged up inside me, where nothing had been before: nothing but the "proper" immersion inside me of a "myself" never identified as this body, still less as this heart, suddenly watching itself. […] From now on it fails, and this strangeness binds me to myself.

Hitherto, his failing heart becomes an intruder, which binds himself to himself. It becomes an intruder occupying a cavern carved up inside his chest. And his intruder, his aching heart, does not merely stay there by itself. It drags him along to the depths of his own body, which was not a place he felt before—not an extended part of his body with tactilely sensed surfaces and areas made of (gently) aching tissues, muscles, organs, bones, tendons, veins and joints. When his heart was silent and concealed—an unnoticed mass—there was no tactilely felt visceral extension to his body, which he now feels as an area of numb or gentle pain. Nancy’s (and Leriche’s) description of the change from a null point to an aching extension captures two possible ways to experience one’s visceral organs tactilely as well as the sudden change between these two modalities: there is a sudden change inside one’s body, which turns one’s attention towards a new area, surface or a place drawn out by touch, movement, ache or pain. Thus, we can understand an intruding organ as a stranger inside one’s own body drawn out by pain. But what (or “who”) exactly is this intruded, “proper”, suffering self?

Nancy’s heart transplant leads to a number of medical procedures, intricate precautions and drugs. And also to cancer, lymphoma, following from necessary and heavy immunosuppressive treatment. All of this leads to experiences of bewilderment and confusion at the heart of how he experiences himself in (or “as”) a relation to “his own” body. Nancy describes how these changes display the strangeness at the very core of the “suffering I”. Nancy writes (2008, p. 169):

Very soon [after immunosuppressive treatment], you are just a wavering, a strangeness suspended between poorly identified states, between pains, between impotences, between failings. Relating to the self has become a problem, a difficulty or an opacity: it happens through evil or fear, no longer anything immediate—and the mediations are tiring.

The empty identity of the ‘I’ can no longer rely on its simple adequation (in its ‘I = I’) as enunciated: ‘I suffer’ implicates two I's, strangers to one another (but touching each other).

As Nancy argues, in suffering “he” becomes “his own” intruder. In suffering, he exists between (i) intimacy of an empty “I” and (ii) his own inescapable suffering and pain felt in distinct areas of his body. These two strange identities touch one another while remaining intrusive to one another. “He” remains stranded between these two facets of himself; he is an “I” who suffers while remaining slightly outside intruding areas demarcated with pain, confusion and discomfort. These painful areas become more clearly refined and distinguishable than his “proper” or “intimate”, yet empty self. He writes (2008, p. 170):

*Corpus meum* [‘my body’] and *interior intimo meo* [‘my innermost inside’], the two being joined, in a complete configuration of the death of god, in order to say very precisely that the subject's truth is its exteriority and its excessiveness: its infinite exposition. The intruder exposes me to excess. It extrudes me, exports me, expropriates me.

This strange and conflicted “self”—Nancy’s *intruder—*offers us a way to elucidate the excruciating experience of being stranded or stuck on sensing one’s own pulsing heart or being stuck in “having to” control one’s breath, when we consider them as intrusive, in the same way as Nancy’s gentle pain, which does not allow any moment to pass without intruding it. But how can we understand the “control”, the ecstatic motility, of organs?

An important aspect concerning the experience of the viscera (in the context of somatic OCD) concerns the nearly total involuntariness of visceral motility. Drew Leder coins this as *I cannot*. Leder’s philosophical position can be broadly described as phenomenological. Leder’s intricate analyses of the viscera (1990) traverse from Descartes, through Husserl and Heidegger to Merleau-Ponty and beyond.^13^ However, a key term for our concurrent analysis of somatic OCD is *ecstasis*, which Leder derives (through Heidegger) from classical Greek. Leder elucidates *ecstatic motility* of living bodies as follows:

This word [*ecstasis*] includes within it the root *ek*, meaning ‘out’, and *stasis*, meaning ‘to stand.’ The ecstatic is that which stands out. This admirably describes the operations of the lived body. The body always has a determined stance—it is that whereby we are located and defined. But the very nature of the body is to project outward from its place of standing. (1990, pp. 21–22).

Leder’s living bodies are distinguished by their ability to move and “project outward”. They live and move *from* the situation and the place they find themselves in the world that they share with other bodies.^14^ His analysis of viscera in the context of *ecstasis* is highly relevant for our discussion of somatic OCD, because his comparison of the viscera and the surface organs in terms of their *volitional motility* (whether or not bodies are able to “project outward” *with* their organs) allows us to distinguish (i) motility obsessions (e.g. breathing) from (ii) tactile obsessions concerning autonomous organs. Leder writes:

The foreignness of this inner body—the automaticity of the ‘it can’, the demanding character of the ‘I must’ [eat, breathe, drink, sleep, etc –JPP]—ultimately refers back to a structure of personal inability. I will term this ‘I cannot’. I cannot act from my inner organs in the way I do from my surface musculature. Though I can lift my arm without any problem, I cannot in the same way choose to secrete a little more bile or accelerate my digestion. (1990, p. 48).

Whereas I can volitionally act *from* or *with* my surface musculature (do things I want to do), I *cannot* volitionally act *from* my visceral body. A heart, stomach or liver lies beyond my volitional control, because I cannot volitionally move, project outward or act *in* the world *from* them or *with* them. Leder’s *visceral inability* seems to define most of our visceral organs: we cannot volitionally act from our hearts, livers or our spleens, which are innervated by the autonomic nervous system. Processes, actions and movements of a heart can be described with Leder’s conceptualisation of *it can* instead. Our lungs are an important exception to visceral inability and I will analyse this further below.

The visceral body is also intriguing, because it places demands on “my body” and on “me”; I must comply with the demands of my visceral body with actions that I perform *with* and through those parts or areas of my body, which I can move, flex or contract volitionally. For example, I must eat and my body reminds me of this need when I get hungry. I also need to breathe and this we will examine below.

## Tactility obsessions

Liver, heart and lungs all share the fact that they are visceral organs situated in the torso. These three organs also demonstrate three different ways we live with our visceral organs, in terms of sensed *tactility* (how we perceive them or with them) and in terms of volitional *motility* (how we can act *from* them or *with* them).

Of these three inner organs, the liver^15^ is the most hidden in the sense that its operations are normally not felt at all. Except for medically well-informed palpations, or due to a numb pain felt in certain hepatic conditions, we do not tactilely^16^ experience the organ in any manner. A liver, therefore, stays concealed; most of the time, we do not feel our livers. The functions of a (non-grafted)^17^ liver are innervated by the autonomic nervous system, which means that the organ does its tasks by itself. Therefore, in Leder’s terms, I *cannot* regulate, manipulate or withhold the actions of my liver according to my wits or wants. Visceral organs innervated by the autonomic nervous system are what makes the automaticity (*it can* in Leder’s vocabulary) of the body and which are unavailable for us in terms of volitional *motility*, unlike the surface musculature such as the arms, lips or toes.

Could we conceive of somatic obsessions concerning the actions, movements, sensations, pains, sounds or processes of the liver? It would be quite hard given the fact that under usual circumstances we neither feel it tactilely (but given a condition such as hypochondria one could feel pain^18^ in the area of the organ) nor can act *from* it. A liver, as it turns out, is not reported in the scarce literature on somatic OCD as an organ with processes that people would become hyper-aware of (unless we include calls of nature as being an obsession concerning our livers). This can be interpreted as being the case due to the unavailability of a liver in terms of its tactility and its motility.

The heart is potentially more present in our awareness than a liver: if I run up a hill or engage in a mindfulness exercise, I can become attentive to the pulse of my heart. I can tactilely perceive my heart in the tissue surrounding it or surrounding my veins. My heart, like my liver, is innervated by the autonomic nervous system, but I can also indirectly affect the processes of my heart through my actions. For example, I can run up a hill or I meditate, which both have an effect on the rate of my pulse. Nevertheless, a heart is still its very own agent in terms of voluntary control; I *cannot* engage in a *direct*^19^ control of my heart in its functions, just as I cannot regulate the operations or actions of my liver. I cannot (literally) act *from* my heart in terms of its functions that are integral to circulatory actions: I cannot volitionally withheld the circulation of my blood, change its direction, open or close various valves, veins, chambers or tubes. Rather, we ought to describe the functions of a heart with Leder’s conceptualisation of *it can.* The anatomical reason for my visceral inability comes down to the fact that it is innervated by the autonomic nervous system.

Somatic obsessions concerning one’s beating heart have been reported by Keuler (2011), and Hershfield and Corboy (2013, p. 177). In these cases, people describe an unbearable condition in which they cannot *not* attend to their pulse—to the extent that their constant experience of their pulse causes them to suffer. In such circumstances, somatic obsessions concerning one’s heart seem to be centred on a temporally constituted felt sense of bodily rhythm, which is regulated by the autonomous neural systems and felt tactilely in the tissue surrounding the organ and one’s veins (and, at times, also in one’s ears). The change from being an unnoticed corporeal feature to being an unbearable aspect of one’s everyday life can be understood with Nancy’s description of how visceral organs become *intrusive* with an experience of pain or movement: at first, a beating heart goes unnoticed. Then, it becomes an extended part of “me,” which “I” touch and which touches “me” from the inside my own body, without letting go. When I suffer from somatic OCD focused on the movement of my heart “I” am, as a “suffering I “, not only ruminating about an intolerable future, but also tactilely stuck into my beating heart, which is intruding my everyday life with its movement.

## Motility obsessions

There is also another type of somatic obsessions, which concerns bodily processes that are partially and, at times, under volitional control. These processes are usually not attended to, but in somatic OCD they become something one is chronically and excruciatingly aware of. Some of the most common reported processes are as follows: breathing, blinking, swallowing of saliva, position of tongue against one’s teeth, etc. In this subsection, I will focus on breathing as an exemplary somatic obsession, which, in addition to being felt *tactilely* in the body, also has to do with the *volitional motility* of bodies.

Leder’s account of visceral automaticity holds true for almost all of the visceral organs, such as the liver, spleen, heart or kidneys: we cannot regulate or withhold their action, yet they place demands with which we must comply if we want to live. However, lungs and breathing seem to differ from other intestinal organs and their autonomy. Breathing usually happens without any conscious input, but such is not always the case, as we know from various therapeutic and non-therapeutic practices, such as mindfulness, yoga, pilates, free diving but also from reports of somatic OCD (Keuler 2011; Hershfield and Corboy 2013). Somatic innervation of lungs does not seem to comply to Leder’s account of visceral inability. He seems to take for granted the automaticity of breathing and, accordingly, he does not question the peculiar status of lungs as non-autonomic visceral organs.

We circulate air with our lungs while we sleep, read or eat. Ventilation of air is an integral part of such a wide array of actions as speaking, singing, coughing or yawning, which are different ways of exhaling warmed air. We can consciously “override” the automaticity of breathing, if we attend to our breath. Some of us can volitionally withhold their breath until they pass out, but not all of us are capable of such a feat. When we breathe, we expand and contract the volume of our lungs, which moves air in and out due to atmospheric pressure.

Leder (1990, p. 50) points out that the “actual” exchange of air in the lungs’ alveolar tissue remains beyond what we can tactilely feel or volitionally control. However, this does not indicate that the activity of breathing would be an autonomic or “vegetative” process; no exchange of air takes place in the alveolar tissue, if the continual and rhythmic movement of ventilation is withheld or interrupted, for whatever reasons. Conversely, breathing is closer to walking than the heartbeat in terms of its innervation (Mitchell et al. 2009). Thus, it seems that we can, in fact, act *from* our lungs in terms of volitional motility, even though our lungs are hidden inside^20^ the chest and most often do their bidding without requiring our engagement at all. In other words, even though breathing is usually “automatic”, it is not an autonomic function. This difference allows us to understand how *motility obsessions* may differ from *tactility obsessions*, which are innervated by the autonomic nervous system. In what I coin as *motility obsessions*, we experience ourselves as attentively stuck in a volitionally innervated process in addition to being “intruded” tactilely by this process that we have volitional control of.

Let us visit two vivid literary descriptions of how we experience our visceral functions in terms of the volitionality of their motility. In *The Lives of a Cell,* Lewis Thomas writes^21^ (1974, p. 78):

If I were informed tomorrow that I was in direct communication with my liver, and could now take over, I would become deeply depressed.

Lewis continues by explaining the reason for his substantial distress in this hypothetical situation: he cannot fathom any of the hepatic decisions made by his liver and prefers not having the slightest responsibility for them. I argue that this is one central aspect of the lived experience of *motility obsessions*: uncertainty regarding the adequate, necessary or optimal performance of a particular bodily process, in terms of what our bodies require. However, if we keep breathing as our exemplary obsessive phenomena, we do not need to merely imagine a “direct communication” with the specific organ(s), because we can, in fact, volitionally control our breath. In this regard, we can understand that one might be agitated about one’s manner of breathing: whether one breathes too fast (as reported by Keuler 2011), too slow, or whether one forgets to maintain one’s breath inadvertently, which might result in tissue damage due to insufficient ventilation. Such distressing experiences of uncertainty are an integral part of the experience of somatic obsessive–compulsive disorder (Keuler 2011), but they do not exhaust the experiential dimensions of the phenomena.

I argue, that there are also other aspects central to the lived experience of motility obsessions. Leder (1990, pp. 47–48, emphasis mine) gives us a clue:

Because I can trust my vegetative body to manage the repetitive assimilations and excretions, I am freed to focus upon novel tasks. *If I had to remember to breathe* or had to stage-manage each phase of my digestion, *there would be little time left for other activities.* The surface body is liberated by such automaticities.

Indeed, if one has to maintain and manage their ventilation for extended periods of times, it can be deeply wearying due to the measure of the task. Such is the experience of somatic obsessive–compulsive disorder, if we look into descriptions of the disorder (Keuler 2011; Hershfield and Corboy 2013, pp. 177–185). I argue that here we have located another pivotal feature inherent to *motility* obsessions, which concerns the experience of being intruded on by one’s own body and no longer having the freedom to live as one pleases. Firstly, we have an experience of the disrupted automaticity of the “vegetative” body, which can be disrupted because breathing is not an autonomic process, we can volitionally breathe. Secondly, we have the inescapable necessity of the process, which manifests itself in the feeling of suffocation, if we fail in volitionally maintaining the process of breathing. In other words, we feel that if we do not consciously maintain the cycle of ventilation, then we are going to suffer, maybe even suffocate, pass out or die. And most of all, we feel discomfort in our lungs and throat. We feel our lungs striving or gasping for fresh air. In effect, the silent automaticity of the ventilation is gone, because we cannot consciously turn away from our ability to act *from* our lungs, in terms of Leder’s account of volitional motility.

Thus, our everyday life is interrupted and intruded by a volitional movement and we feel trapped by having to maintain it, because we can turn away neither from:

(i) Being able to breathe volitionally;
(ii) Having to breathe constantly.

This experience has previously been typified as “magnification of the thought” or as “catastrophizing about an intolerable future” (Hershfield and Corboy 2013, p. 181) in the previous analyses of the condition. However, if we consider the lived experience of constantly having to manage one’s breath in order not to experience the consequences of insufficient ventilation, the intrusion of one’s life is not limited to the region of thinking; it comes down to how we tactilely experience our lungs while we breathe, how it feels to move one’s abdomen in order to breathe and how it feels to suffocate when one does not continue breathing. Fearful thoughts, critical rumination, and catastrophizing may follow from the experience of a disrupted automaticity of ventilation, but such cognitive aspects of OCD do not adequately describe the central role of the body in the experiential dimensions of motility obsessions. If I feel that I have to maintain my breathe, I suffer because I feel *intruded* by the volitional motility of my lungs.

## Conclusions

I have argued above that in both tactility obsessions and motility obsessions, we are not only ruminating or thinking about an intolerable future following from not being able to steer our attention away from our bodily processes. Rather than consisting merely in unusual thoughts, the intrusive experience has to do with how we tactilely feel our bodies and how it feels when we volitionally move with our bodies.

In the case of tactility obsessions, any rumination of an intolerable future follows from what we sense *in* and *with* our bodies. At first, an unnoticed organ, e.g. my heart, is absent or concealed. Then, it becomes an extended part of “me,” which “I” touch and which touches “me” from the inside my own body, without letting go. When I suffer from somatic OCD focused on the movement of my heart “I” am, as a “suffering I “, not only ruminating excessively about an intolerable future, but also feel tactilely stuck into my beating heart, which is intruding my life with its movement.

When I suffer from a motility obsession, I feel stuck on a necessary bodily process, which I have volitional control over. Fearful thoughts may follow from the experience of disrupted automaticity of such a bodily process, but cognitive aspects of OCD do not adequately describe the central role of the body in the experience of motility obsessions. If I feel that I have to maintain my breathe, I suffer because I feel intruded by the volitional motility of my own lungs.

## Footnotes

1 Or in other sources, “sensory”, “somatoform”, “hyperawareness”, or “sensorimotoral” obsessive–compulsive disorder.

2 Hereinafter “somatic OCD”.

3 Greek *hyper*: over, above, beyond, excessive, exceeding.

4 See Bürgy (2019), for an excellent overview of developments in our understanding of obsessive–compulsive disorder organised according to a framework developed by Karl Jaspers through his seven different editions of “General Psychopathology”.

5 Concerning the dimensions of obsessions and compulsions in OCD, see: Abramowitz et al. (2010), Denys et al. (2004).

6 The question concerning how clearly defined and separate are the dimensions labelled as “obsessions” and “compulsions” falls beyond the scope of this article. Cf. Bürgy (2019).

7 I am aware of the discussion in clinical psychology concerning the question of “subtype vs spectrum” in OCD, but this discussion falls beyond the scope of this paper. Cf. Taylor (2010), Hollander et al. (2012).

8 Keuler (2011), Hershfield and Corboy (2013).

9 For an excellent summary of corporeal transparency, see Fuchs (2005).

10 As we may remember, Rene Leriche‘s definition of health is life lived in the silence of the organs. Nancy attributes this definition to French surgeon Xavier Bichat in Nancy (2008) for unknown reasons. Leriche’s definition has intriguing implications for understanding sickness and pain metaphorically as sonorous phenomena. For an excellent overview of Leriche see Canguilhem (1991, p. 91–102).

11 There is also a temporal aspect to the absence of one’s own heart, which Nancy does not analyse with the same rigour he warrants to the felt sense of intrusion. An absent organ does not turn us towards the withering of our finite bodies and organs. An unnoticed heart is a transparent and non-extending mass, but it also beats endlessly and infinitely.

12 Questions concerning the “voice” of organs go beyond the scope of this text. The most obvious non-metaphorically noisy organ is a ringing ear.

13 Here we cannot delve into *The Absent Body* systematically nor extensively; in this short text I am focusing on Leder’s conceptualisation of the visceral inability as a conceptual addition to Nancy’s *intruder.*

14 In other words, Leder’s bodies find themselves *thrown* in the world, which is an existential structure analysed extensively by Heidegger in *Being and Time*.

15 For an intriguing account of an experience of liver abnormality and deterioration, see Varela (2001).

16 In this text, I choose to exclude the realm of somatic sounds from my analysis. Heart and lungs are also sonorous organs.

17 A grafted liver is neurally isolated from one’s body and it does (remarkably, if you ask me) its biddings by itself: “Following liver transplantation, all hepatic nerves are transected; thus, liver allografts are completely isolated from neural control of their hosts. Despite this absolute denervation, liver allograft function does not appear to be significantly impaired after successful transplantation”, Colle et al. (2004, p. 924).

18 An analysis of the similarities between somatic obsessions, the felt sense of chronic pain and the experience of tinnitus would move beyond the scope of this article. For discussion of pain, see: Scarry (1985).

19 Although indirect control of heart’s beat through meditation, relaxation or breathing is possible, it does not change the fact that one cannot volitionally flex the muscles of their heart.

20 Although one could argue that bodily surface continues into cavernous lungs.

21 Also cited by Leder in *The Absent Body*, 48.
